# Exploring the Impact of Short Term Travel on Gut Microbiota and Probiotic Bacteria Mediated Stability

**DOI:** 10.3390/biomedicines12071378

**Published:** 2024-06-21

**Authors:** Yiming Zhao, Chunyan Li, Kaijuan Wu, Hao Chen, Qingqun Wang, Ying Xiao, Siqi Yao, Ao Hong, Man Zhang, Shibo Lei, Wenyu Yang, Shukun Zhong, Abdulrahim Umar, Jing Huang, Zheng Yu

**Affiliations:** 1 Human Microbiome and Health Group, Department of Microbiology, School of Basic Medical Science, Central South University, Changsha 410013, China; yimingzhao970904@163.com (Y.Z.); fclichunyan@126.com (C.L.); chenho998@163.com (H.C.); 17803210938@163.com (Q.W.); xy18373160437@163.com (Y.X.); ysqzj1999@163.com (S.Y.); 226511010@csu.edu.cn (M.Z.); lei2102034@163.com (S.L.); uabdulrahim@csu.edu.cn (A.U.); 2 Human Microbiome and Health Group, Department of Parasitology, School of Basic Medical Science, Central South University, Changsha 410013, China; wkjcsu@csu.edu.cn (K.W.); 226511066@csu.edu.cn (A.H.); 15234435723@163.com (W.Y.); zhongsk15799@163.com (S.Z.); jing_huang@csu.edu.cn (J.H.)

**Keywords:** travel, gut microbiota, probiotic bacteria, microbial disorder

## Abstract

Although travelers are frequently accompanied by abdominal discomfort and even diarrhea, not every trip can cause this issue. Many studies have reported that intestinal microbes play an important role in it. However, little is known about the reason for the dynamics of these intestinal microbes. Here, we delved into the effects of short-term travel on the gut microbiota of 12 healthy individuals. A total of 72 fecal samples collected before and after one-week travel, alongside non-traveling controls, underwent amplicon sequencing and a series of bioinformatic analyses. We found that travel significantly increased intra-individual gut microbiota fluctuations without diarrhea symptoms. In addition, the initial composition of the gut microbiota before travel emerged as a crucial factor in understanding these fluctuations. Travelers with stable microbiota exhibited an enrichment of specific probiotic bacteria (*Agathobaculum*, *Faecalibacterium*, *Bifidobacterium*, *Roseburia*, *Lactobacillus*) before travel. Another batch of data validated their predictive role in distinguishing travelers with and without the gut microbial disorder. This work provided valuable insights into understanding the relationship between gut microbiota and travel. It offered a microbiota-centric perspective and a potential avenue for interventions to preserve gut health during travel.

## 1. Introduction

The human intestine harbors a diverse microbial community known as the intestinal microbiota, which has co-evolved with its host over millions of years [1]. This community, collectively referred to as gut microbiota, plays a crucial role in maintaining human health and is intricately involved in the entire process of human growth [2]. It protects the body by strengthening the integrity of the intestinal barrier [3,4], providing resistance against pathogenic microbes [5], and regulating the immune system [6,7]. 

The disruption of gut homeostasis can have detrimental effects on the human body, leading to gastrointestinal discomfort and even illness. This disruption, known as gut microbial dysbiosis, can occur due to potentially harmful intestinal microbiota or the absence of beneficial ones [8]. Increasingly, people recognize that changes in the composition and functional potential of intestinal microbiota can impact host health and contribute to the development of various diseases, including obesity, metabolic syndrome, and inflammatory diseases [9,10].

The environment plays a significant role in influencing human gut microbiota [11]. Travel, with its associated environmental changes, is a complex process. It can cause some of the original symbiotic microbes in the gastrointestinal tract to be unable to adapt to the new environment, disrupting gut homeostasis. Physiological changes in the body before and after travel can be considered triggers for certain discomforts, such as diarrhea [12]. Furthermore, even non-diarrheal travelers exhibit changes in the composition of their gut microbiota [13]. Langelier et al. [14] revealed that travel caused an increase in antimicrobial resistance genes and a higher proportion of gut *Escherichia* species. Similarly, Kampmann et al. [15] also reported that the relative abundance of *Enterobacteriaceae* bacteria, often associated with infection, inflammation, and antibiotic resistance, was dramatically elevated after travel. Conversely, there is a remarkable decrease in the relative abundance of health associated gut microbes.

However, not all individuals are affected by travel related disruptions in their intestinal microbes. David et al. [16] conducted research, tracking the commensal microbial communities of two individuals with different lifestyles over a year, and found that the differences between individuals were much more significant than the differences within individuals. Different people exhibit varying responses to travel [17]. While some individuals experience noticeable disruptions in their gut microbiota, others are only minimally affected with no significant alterations. The influence of travel on gut microbes varies from person to person. The reason behind these individual differences in gut microbial response is a critical question in understanding the relationship between travel and gut microbiota. However, the precise reasons are not yet fully understood. In this study, we collected 72 fecal samples longitudinally from 12 people with short term travel (7 days) and those who had not traveled at all. Amplicon sequencing was performed to track the dynamics of the gut microbiome, and the results demonstrated that travel promotes alterations in the gut microbial composition. In addition, several travelers did not experience gut microbiota dysbiosis, and our analysis, combining our data with another set of metagenomic sequencing data from other travelers using a machine learning approach, revealed a relationship between these outcomes and certain common probiotics. This work will provide new insight into understanding the role of gut microbiota in travel and gut health.

## 2. Materials and Methods

### 2.1. Participant Recruitment and Sample Collection

The trial was approved and registered by the institutional review board of the school of basic medical science, Central South University (No. 2021-KT75). Referring to the research method of Manish Boolchandani et al. [18], we recruited students who were enrolled at the Central South University, and all participants signed an informed consent form. Because the subjects of the study are healthy individuals, we ensured the participants have no known health problems that may affect the intestinal microbiota. In detail, if participants took antibiotics in the last three months or had a history of inflammatory bowel disease, diabetes or other potential diseases, they were excluded from the study cohort. In addition, all participants agreed to complete a standardized enrollment questionnaire that collected baseline demographic and epidemiologic data, diet habits, and medical and travel history (Table S1). Based on the questionnaire result, we scored the diet difference of each participant from 1 (exactly the same) to 5 (totally different). The distance and trajectory of the trip were counted according to the information provided by the participants. Finally, we included a total of 12 healthy participants who had a similar short term travel history in this study.

We provided all participants with sterile stool collection kits and instructions on collecting a stool sample with their bowel movements. The participants collected their first stool samples in the morning. Samples were collected at two time points: one week before the trip and one week after the trip. Three samples of defecation were collected at each time point. We gave the fecal samples a stool grade based on the Bristol stool form scale (BSS) of stool consistency [19], ranging from grade 1 (normal, hard) to grade 7 (loose, diarrheal). For participants without travel, we also took 6 samples from each person at the same two time points. We saved the collected fecal samples and immediately placed them in a −80 °C freezer for the next steps.

### 2.2. DNA Extraction and 16S Ribosomal RNA Gene Sequencing

We accessed the fecal samples through 16S *Ribosomal* RNA (rRNA) gene sequencing [20]. In detail, DNA Extraction Total genomic DNA samples were extracted using the OMEGA DNA Kit (M5635-01) (Omega Bio-Tek, Norcross, GA, USA), following the manufacturer’s instructions. The PCR amplification of the bacterial 16S rRNA genes V3–V4 region was performed using the forward primer 338F (5′-ACTCCTACGGGAGGCAGCA-3′) and the reverse primer 806R (5′-GGACTACHVGGGTWTCTAAT-3′) [21]. Sample-specific 7 bp barcodes were incorporated into the primers for multiplex sequencing. The PCR components contained 5 μL of buffer (5×), 0.25 μL of Fast pfu DNA Polymerase (5 U/μL), 2 μL (2.5 mM) of dNTPs, 1 μL (10 μM) of each forward and reverse primer, 1 μL of DNA Template, and 14.75 μL of distillation-distillation H_2_O (ddH_2_O). Thermal cycling consisted of an initial denaturation at 98 °C for 5 min, followed by 25 cycles consisting of denaturation at 98 °C for 30 s, annealing at 53 °C for 30 s, and extension at 72 °C for 45 s, with a final extension of 5 min at 72 °C. PCR amplicons were purified with Vazyme VAHTSTM DNA Clean Beads (Vazyme, Nanjing, China) and quantified using the Quant-iT PicoGreen dsDNA Assay Kit (Invitrogen, Carlsbad, CA, USA). After the individual quantification step, amplicons were pooled in equal amounts, and pair-end 2 × 250 bp sequencing was performed using the Illumina NovaSeq platform with NovaSeq 6000 SP Reagent Kit (San Diego, CA, USA) (500 cycles).

### 2.3. 16S Ribosomal RNA Gene Sequencing Data Processing and Analysis

For 16S rRNA gene sequencing data, we performed a series of processes on the raw sequence data. Firstly, the primers of sequence data were trimmed with Cutadapt [22] (version 3.4), and the sequences were merged and truncated to 400 bp using Vsearch [23] (version 2.7.0). We performed quality control in Qiime2 [24] (version 2021.4.0) with the ‘quality-filter’ function by default parameters. Next, the clean reads were chimera filtered, denoised, clustered to the Operational Taxonomic Unit (OTU) and profiled with the ‘deblur’ function in Qiime2. To reduce false positives in obtaining data, only the OTUs that mapped reads of more than two were retained and rarefied to the minimum read counts using the R package ‘picante’ [25] (version 1.8.2). Meanwhile, the representative sequence was selected from each OTU to perform taxonomic classification by the feature-classifier function in Qiime2 against the Silva database [26] (version 138). The Shannon-Wiener index was calculated to investigate the alpha-diversity of microbial communities across samples. At the same time, the Bray–Curtis (BC) distance metrics were calculated based on the relative abundance of OTUs and visualized via nonmetric multidimensional scaling (NMDS) by R package ‘vegan’ [27] (version 2.5.7). In addition, the ‘envfit’ function in it was used to perform a regression of the coordinates with the microbial factors. At this time, the microbial factors are taken as the response variables, and the coordinates are taken as the explanatory variables for linear regression.

### 2.4. Public Data Collection and Validation Analysis

To validate the universality of initially enriched microbes in travelers without the disorder, an additional cohort of travelers with shotgun metagenomic sequencing data was collected in the study published in 2022 [18]. According to the metadata, we filtered and downloaded 19 samples (NCBI accession ID: SRR13646884, SRR13646939, SRR13646943, SRR13646966, SRR13646981, SRR13646984, SRR13647282, SRR13647418, SRR13647494, SRR13647523, SRR13646912, SRR13646958, SRR13647156, SRR13647257, SRR13647376, SRR13647401, SRR13647431, SRR13647534, and SRR13647554) from the non-diarrhea people under ’week 0′. Because these samples are collected on the first day after the destination, they can represent the initial gut microbial composition before travel. Then, metaphlan [28] (version 4.0.6), which is based on mapping reads of unique marker genes was performed to obtain each sample microbial profile. In addition, we checked the metadata of this batch of data and performed area under curve (AUC) analysis using the R package ‘pROC’ [29] (version 1.17.0.1).

### 2.5. Other Statistical Analysis

For parametric feature-wise multivariable testing, we used MaAsLin2 with default parameters [30], which finds associations between microbial relative abundance and BC distance. MaAsLin2 can utilize a transformed generalized linear model to associate each feature iteratively with covariates. With the default settings, MaAsLin2 implementation uses a log-transformed linear model on Total Sum Scaling (TSS) normalized quality controlled data.

The baseline data were obtained using the R package ‘compareGroups’ (version 4.5.1) [31]. It can automatically perform a Shapiro-Wilks test to determine whether the distribution is normal or abnormal. And, depending whether the variable is considered as continuous normal distributed, the *t*-test or Kruskall-Wallis test are performed, respectively. If the variable is categorical, the chi-squared test is performed. All other statistical tests used the ‘scipy’ or ‘statsmodels’ package in Python. Most of the data visualization was applied using ‘ggplot2’ in R or ‘matplotlib’ in Python.

## 3. Results

### 3.1. Overview of the Travel and No Travel Cohorts

**Figure 1:**
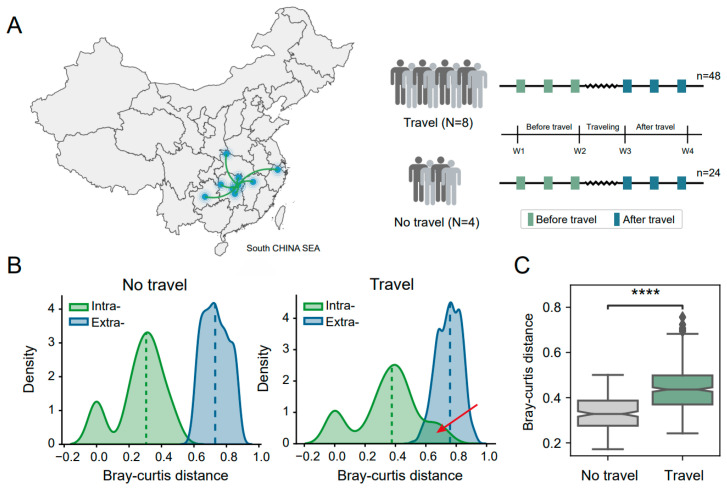
Study design and the significant alteration of gut microbiota in the cohort of travelers. (**A**) Overview of the study design. The map on the left shows the travel trends of all travelers, and the workflow schematic diagram on the right illustrates the details of sample groups and data collection. (**B**) The distribution of Bray–Curtis distance based on the gut microbial relative abundance in two groups. ‘Intra-’ and ‘Extra-’ represent the distances within and between individuals, respectively. (**C**) The Bray–Curtis distance is used to estimate the alteration of gut microbiota (Pairwise comparison between samples of ‘after travel’ and ‘before travel’) between two groups (two-sided Mann–Whitney–Wilcoxon test, **** *p*-value < 0.0001).

We assembled two cohorts (Travel and No travel) of 12 students (75% female, 25% male) attending the Central South University of Changsha, Hunan, China, and all participants stayed in Changsha for at least six months before traveling. Stool samples were collected within one week before traveling, and additional samples were collected after traveling. The eight individuals who traveled (Travel group) went on one-week trip. The traveling was within or across provinces with a travel distance of about 115 km to 860 km. We collected 72 stool samples, with three samples per individual (Figure 1A). We collected detailed demographics, medical history, and other information from each participant, and cohort characteristics. A summary of metadata features is listed in Table S2. No significant differences existed between the Travel and No travel groups for sex, age, BMI, and smoking status (Table S2). No diarrhea (BSS score > 4) was reported from all participants during the sampling period. To characterize the gut microbial communities of the travelers, we performed 16S rRNA gene sequencing on the 72 stool samples and generated a taxonomic composition. 

### 3.2. Travel Promoted the Fluctuation of the Intestinal Microbiome

The intestinal microbiome followed a dynamic process, also reflected in our results; the intestinal microbiota within an individual (Intra-) changes with time. However, it is noteworthy that the fluctuation of microbiomes intra-individually over time was increased in the ‘Travel’ group, from about the median Bray–Curtis (BC) distance of 0.25 to 0.4, more than 1.5 times higher than the ‘No travel’ group. Moreover, this level of microbial alteration could even reach the threshold of the size among different individuals (Extra-) (Figure 1B). To compare the level of intestinal microbial alteration in the ‘Travel’ and ‘No travel’ groups, we estimated the BC distances between W1–W2 (the samples collected before the traveling week) and W3–W4 (the samples collected in the time after the traveling week). Not surprisingly, the level of altered difference in microbial composition in the former group was significantly less than the latter (Figure 1C, two-sided Mann–Whitney–Wilcoxon test, *p*-value < 0.0001), which indicated that the travel promoted the fluctuation process of gut microbiota.

**Figure 2:**
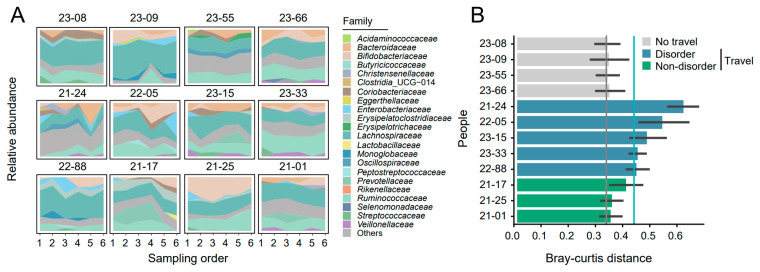
Gut microbiota dynamics during traveling. (**A**) The gut microbial composition dynamics among different people at the family level. Only the top five taxa are shown in each sample, and the remaining ones are grouped as ‘Others’. (**B**) The Bray–Curtis distance (after travel versus before travel) in the different people and the grey and blue lines represent the median values in the two groups (No travel and Travel). According to the median values, the people in the ‘Travel’ group are divided into ‘Disorder’ and ‘Non-disorder’ sub-groups.

Although the initial gut microbiome in participants from the ‘No travel’ group differed, the gut microbiota seemed stable. As for the participants from the ‘Travel’ group, heterogeneity was observed in the alteration of gut microbiome (Figure 2A). For example, after traveling, the relative abundance of *Bacteroidaceae* in participant ‘21–24; was increased; however, it was decreased in the participant ‘22–05’. This suggested that the fluctuation of gut microbiota affected by travel was complex.

### 3.3. The Variable Level of Gut Microbiota Alteration in Different Travelers

According to the BC distances calculated by the microbial composition data in the W1–W2 and W3–W4, almost all individuals in the ‘No travel’ group shared an average BC distance of about 0.35 (Figure 2B). Therefore, we selected a BC distance of 0.35 as the baseline for normal dynamic variation. In the ‘Travel’ group, almost every individual had an average BC distance of more than 0.35, consistent with the result that travel could promote gut microbial fluctuation. However, we found that the level affected by travel was variable in different people. The intestinal microbes of several individuals (such as the participant ‘21–01’) in the ‘Travel’ group did not appear to be affected by travel (Figure 2A,B). To further explore the potential reason for this difference in different people, we sub-divided travelers into ‘Disorder’ (21–24, 22–05, 23–15, 23–33, and 22–88) and ‘Non-disorder’ (21–17, 21–25, and 21–01) groups according to the median BC distance in the Travel group, and the individuals from the ‘Non-disorder’ group accounted for 37.5% of all travelers. Besides, in another study [18], nearly half of the participants (47.4%) were also not affected by travel according to the definition in (Figure S1), which emphasized the variation in gut microbiota alteration among different individuals. 

### 3.4. Unique Initial Intestinal Microbiota in Travelers without the Gut Microbial Disorder

**Figure 3:**
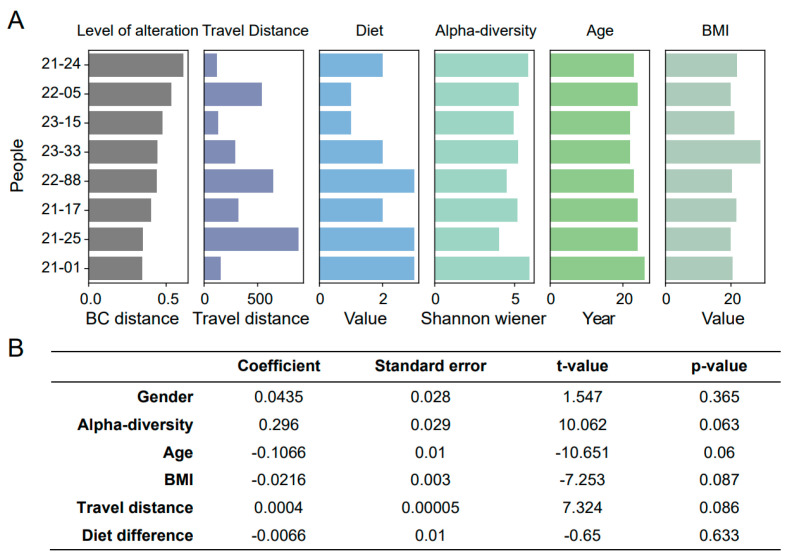
The correlation between multiple individual factors and the level of altered gut microbiota. (**A**) From left to right, the level of altered gut microbiota, travel distance, diet difference, alpha-diversity, age and BMI in different travelers are shown. (**B**) The multiple linear regression is performed to determine the correlation between several factors and the level of altered gut microbiota.

Travel is a comprehensive process, and various factors, such as diet [32], can affect the composition of intestinal microbes during travel. Therefore, we first checked several factors (travel distance, diet, gender, BMI, age, and alpha-diversity) to explore the potential reasons for varying gut microbial alteration in different travelers (Figure 3A). The results showed that these factors did not significantly correlate with this individual difference (Figure 3B). Therefore, we speculated that the initial gut microbiota may play a role before travel. 

**Figure 4:**
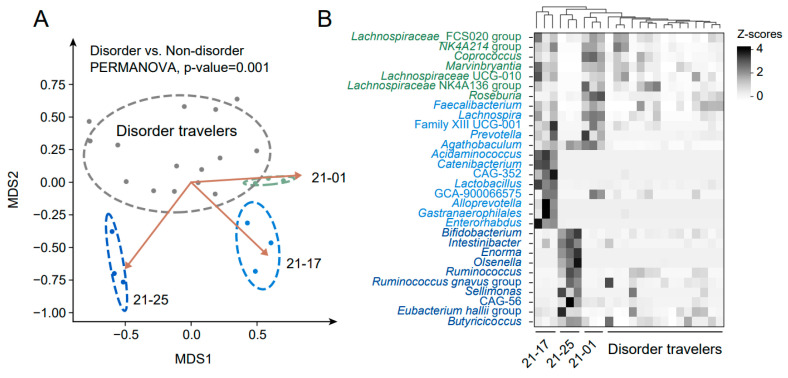
Unique initial gut microbiota in the non-disorder travelers. (**A**) The NMDS analysis of initial gut microbiota (before travel) from travelers. For visualization, all fitted genus features (FDR < 0.05, R^2^ > 0.3) of non-disorder people were integrated into three arrows in the ordination. The top of the figure shows the *p*-value of genus composition under different sub-groups using the PERMANOVA test. (**B**) The heatmap plot showed the relative abundance of the corresponding fitted genus for three non-disorder travelers, and the genus color was consistent with the color in (**A**).

**Figure 5:**
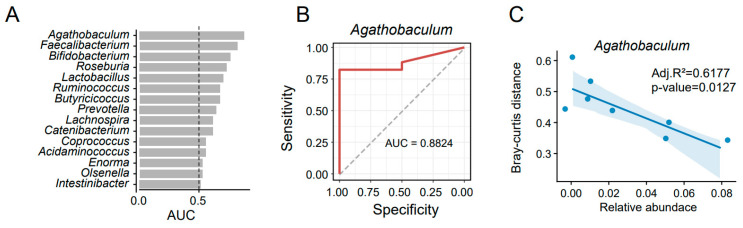
Validation of the probiotic bacteria in maintaining the gut microbial stability with other public data. (**A**) All genera fitted to non-disorder travelers are measured by area under the curve (AUC) values using the public dataset, and the receiver operating characteristic curve (ROC) of *Agathobaculum* was shown in (**B**). (**C**) The correlation between the relative abundance of genus *Agathobaculum* and Bray Curtis distance, which measured for the level of gut microbial alteration.

The higher initial alpha-diversity does not make the gut microbiota more stable under travel intervention (Figure 3A). However, we found that the initial composition of gut microbiota in non-disorder participants was significantly different from that in the disorder group according to the analysis of NMDS (Figure 4A, PERMANOVA, *p*-value = 0.001). Therefore, the initial gut microbiota may be related to different travelers’ differences in microbial alteration. There were 30 kinds of gut microbes detected to enrich in the non-disorder group (R > 0.3 and FDR < 0.05). Although the gut microbes in these three participants (21–25, 21–17, and 21–01) were inconsistent, most enriched microbes were probiotic bacteria, such as *Bifidobacterium* and *Ruminococcus* (Figure 4B). Afterwards, another batch of shotgun metagenomics sequencing data [18] was introduced to check the accuracy of these enriched microbes using a machine learning method (Figure S2). Despite part of the enriched microbiota not mapping the uniform taxonomical name, almost all checked enriched microbes had an area under curve (AUC) value of more than 0.5, which meant that the predicted potential of using these enriched microbes (Figure 5A) was accurate. Among them, genus *Agathobaculum* had the highest AUC value (0.8824, Figure 5B). In addition, *Agathobaculum* was the only genus with a negative correlation with BC distance between the gut microbiota before and after travel (Figure 5C), and determined as the related taxonomic markers by MaAsLin2, which is a model to study with longitudinal designs. These results highlighted the gut microbiota’s potentially beneficial function to protect the gut microbial community from the interference of travel.

## 4. Discussion

Here, we reported a longitudinal study on the differential response of gut microbes to short-term travel in healthy people. We observed that the level of intestinal microbial alteration of volunteers with travel was significantly larger than those of non-travelers, and different travelers responded variously to the travel. We investigated a series of reasons, and the results showed that the initial microbial composition before travel was associated with this individual difference; further analysis revealed that some probiotic bacteria may play a specific role in it. Another batch of shotgun metagenomics sequencing data from a more comprehensive source cohort was used to construct and validate a machine learning model with high accuracy and an accurate recall rate. This emphasized the potential role of these probiotic bacteria in maintaining gut homeostasis.

Our results showed that the gut microbes of healthy individuals were not unchangeable; they would fluctuate with time, which was consistent with previous studies [33]. In addition, there was diversity in the healthy human gut microbiota [34]. However, travel promotes or rushes the process of gut microbial fluctuation, and travel can contribute to the global spread of antimicrobial resistance and diarrhea [14,35,36]. One interesting finding was that although all travelers did not experience diarrhea, a higher risk of gut microbial alteration was found in travelers than non-travelers in our study. Short-term travel may be why 37.5% of travelers had little change in intestinal microbes. However, another study found that travel did not affect nearly half of the travelers’ intestinal microbiota [14]. This difference in intestinal microbes’ response from different people to travel deserves attention. Therefore, we included a non-traveling control cohort in the same period, which helped identify the subtle changes of intestinal microbes more sensitively and selected the best threshold for gut microbial disorder. 

Next, we tried to explain this kind of individual difference. However, no significantly correlative known external factors were found in this study. Travelers were contacting an altered environment, with many covariates that caused this promotion of microbial fluctuation, such as mood [35], diet [36] and so on. The factors that led to gut microbial disorder were complex and ambiguous. As for gut microbial diversity, there were contradictions in some studies; for example, some studies reported that the microbial diversity did not vary with travel [15,37], while the high diversity was related to the intestinal stability of travelers in Langelier et al.’s research [14]. Our result was consistent with the former; we had not found that higher initial alpha-diversity made gut microbiota less affected by travel. However, compared to travelers with noticeable gut microbial change by travel, the initial gut microbial composition in non-disorder travelers before travel showed significant specificity, enriched by probiotic bacteria.

Probiotic bacteria had a positive role in maintaining intestinal stability and health [38,39], and our results were consistent with that. These probiotic bacteria were validated by combining another batch of data with a machine-learning method. Genus *Agathobaculum*, *Faecalibacterium*, *Bifidobacterium*, *Roseburia* and *Lactobacillus* were the top five gut microbes, with the best predictive ability to distinguish whether the traveler will have a gut microbial disorder. Among them, *Agathobaculum* [40], *Faecalibacterium* [41,42] and *Roseburia* [43,44] were reported as the producers of short-chain fatty acids (SCFAs). SCFAs had a crucial role in gut physiology and host wellbeing. It is the main energy source for the colonocytes, and it has protective properties that maintain intestinal health [45]. In addition, *Faecalibacterium* is the core bacterium in healthy people’s gut that enhances the intestinal barrier function and affects paracellular permeability [46]. It is a next-generation probiotic bacteria, affecting gastrointestinal diseases or disorders [47]. Genus *Bifidobacterium* and *Lactobacillus* were common probiotic bacteria incorporated into many functional foods as active ingredients, and they were considered to affect colon regularity [48,49,50].

Nevertheless, there are some limitations in our study. For instance, the differences of initial gastrointestinal motor functions [51] in different individuals may compromise the results. Although we introduced other public data to validate our results, the larger clinical cohorts need to be covered in the future. 

## 5. Conclusions

In total, based on the gut microbiome data from two cohorts (Travel and no travel), we found travel would promote the fluctuation of the intestinal microbiota, and different individuals had different responses. We found the enrichment of probiotic gut microbiota in travelers without the gut microbial disorder. In addition, this discovery was verified by other public data. These results showed the potential of probiotic bacteria to maintain the intestine’s health and protect the gut microbial community from the interference of travel. This study provides valuable insights into the complex interactions between travel and gut microbiota, and provides the potential reasons for explaining the difference in gut microbial alteration among different travelers. Further research in this direction holds the potential to uncover novel therapeutic targets and strategies for promoting traveler health through microbiota modulation.

## Data Availability

The raw sequence data reported in this paper have been deposited in the Genome Sequence Archive [52] in the National Genomics Data Center [53], China National Center for Bioinformation/Beijing Institute of Genomics, Chinese Academy of Sciences, under accession number CRA014598, and are publicly accessible at https://bigd.big.ac.cn/gsa (accessed on 23 January 2024).

## Supplementary Materials

The following supporting information can be downloaded at: https://www.mdpi.com/article/10.3390/biomedicines12071378/s1, Figure S1: The proportion of ‘No disorder’ among all 19 healthy travelers in another public cohort. Figure S2: The public cohort overview, sample filtering, and exploration using another public data. Table S1: The standardized enrollment questionnaire. Table S2: Charac-teristics of all participants.
