# Supplementary material for: Exploring the Impact of Short Term Travel on Gut Microbiota and Probiotic Bacteria Mediated Stability
Source: Biomedicines. 2024 Jun 21;12(7):1378. doi: 10.3390/biomedicines12071378 (PMC11274169; doi:10.3390/biomedicines12071378)
Supplement: Supplementary file 1 [file biomedicines-12-01378-s001.zip › Supplementary Figures.pdf]

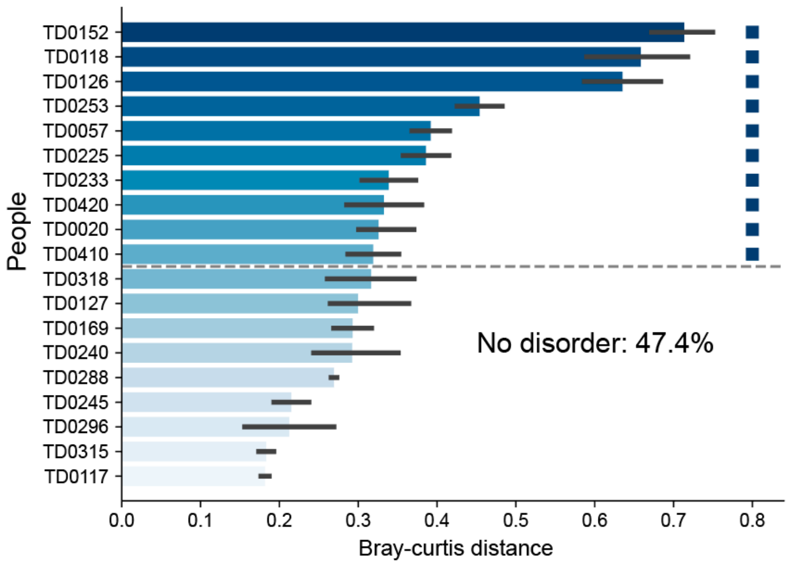

Figure S1. The proportion of 'No disorder' among all 19 healthy travelers in another public cohort. The X-axis showed Bray-Curtis dissimilarities between each subjects' samples and their 1st-week baseline sample. And the people marked with blue squares means their level of gut microbial alteration by travel is higher than the threshold of gut microbial disorder.

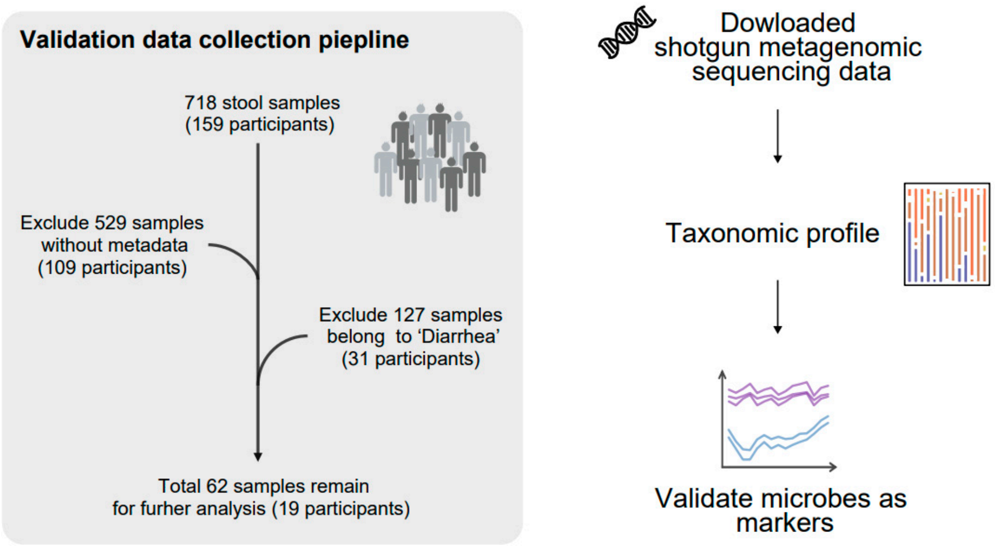

Figure S2. The public cohort overview, sample filtering, and exploration using another public data.
