# Supplementary material for: Exploring the Impact of Short Term Travel on Gut Microbiota and Probiotic Bacteria Mediated Stability
Source: Biomedicines. 2024 Jun 21;12(7):1378. doi: 10.3390/biomedicines12071378 (PMC11274169; doi:10.3390/biomedicines12071378)
Supplement: Supplementary file 1 [file biomedicines-12-01378-s001.zip › Table S1.pdf]

Table S1 The standardized enrollment questionnaire

Basic information

ID:

Gender:

Age:

Height (cm):

Weight (Kg):

Smoking or not:

Did you use antibiotics in the past three month:

Time from last departure from Changsha:

Travel destinations (please separate them with commas if there are more than one):

Planned travel days:

Planned mode of travel (plane/train/high-speed rail/bus/self-driving):

Eating habits

1. Will you keep the same routine during your trip?

() always () almost always () sometimes () rarely () never.

2. Do you overeat when you meet your favorite food?

() always () almost always () sometimes () rarely () never.

Do you wash your hands before eating?

() always () almost always () sometimes () rarely () never.

4. What do you think is the difference between the changes of your diet structure at your destination and those before your trip?

() Totally different () Very different () Little different () Almost the same () Exactly the same.

5. What are your dietary preferences?

() I like fried chicken, hamburgers, pizza and other tasty foods, but I don't like eating fruits and vegetables very much.

() There must be vegetables and meat at every meal, and the ratio of the two is almost the same.

() Mainly vegetarian, occasionally eating a little meat/eggs.

() Don't eat meat or fried food at all, a fanatical vegetarian.

() None of the above, please write down your eating habits:

Other

Did you feel unwell during your absence from Changsha? If so, please record the time and place, symptoms of physical discomfort and drug use:

Date of completion: _ _ _ _ _

Date of submission: _ _ _ _ _
