# Supplementary material for: Exploring the Impact of Short Term Travel on Gut Microbiota and Probiotic Bacteria Mediated Stability
Source: Biomedicines. 2024 Jun 21;12(7):1378. doi: 10.3390/biomedicines12071378 (PMC11274169; doi:10.3390/biomedicines12071378)
Supplement: Supplementary file 1 [file biomedicines-12-01378-s001.zip › Table S2.pdf]

Table S2 Characteristics of all participants

	No travel	Travel	p-value
	N=4	N=8	
Gender:			0.236
Gender (Female)	2 (50.0%)	7 (87.5%)	
Gender (Male)	2 (50.0%)	1 (12.5%)	
Age	23.8 (2.06)	23.5 (1.31)	0.835
BMI	20.4 [20.0;20.9]	20.6 [20.2;21.6]	0.798
No smoke	4 (100%)	8 (100%)	1

All participants did not take antibiotics for three months.
